# Supplementary material for: PD-L2 suppresses T cell signaling via coinhibitory microcluster formation and SHP2 phosphatase recruitment
Source: Commun Biol. 2021 May 14;4:581. doi: 10.1038/s42003-021-02111-3 (PMC8121797; doi:10.1038/s42003-021-02111-3)
Supplement: Supplementary file 2 — Supplementary Information [file 42003_2021_2111_MOESM2_ESM.pdf]

Supplementary Fig. 1

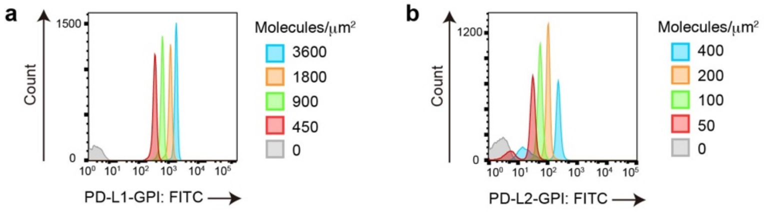

Supplementary Fig. 1 Expression of PD-1 ligands on silica beads.

Silica beads were coated by lipid-bilayers containing mPD-L1–GPI (a) or mPD-L2–GPI (b) at the indicated densities. The expression of mPD-L1 and mPD-L2 on the beads was analyzed by FACS with FITC-labeled anti-PD-L1 and anti-PD-L2, respectively.

Supplementary Fig. 2

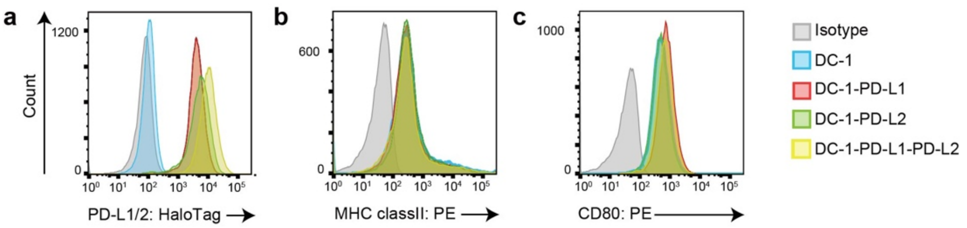

Supplementary Fig. 2 Normalization of the expression of PD-L1 and PD-L2 on DC-1 cells. DC-1 cells were transduced with either HaloTag-tagged mPD-L1, mPD-L2 or both. The expression of mPD-L1– or mPD-L2–HaloTag on DC-1 cells was analyzed by FACS with TMR-labeled HaloTag ligands (a). The expression of MHC Class II (b) and CD80 (c) on these DC-1 cells was analyzed by FACS with PE-labeled anti-MHC Class II and anti-CD80, respectively.

Supplementary Fig. 3

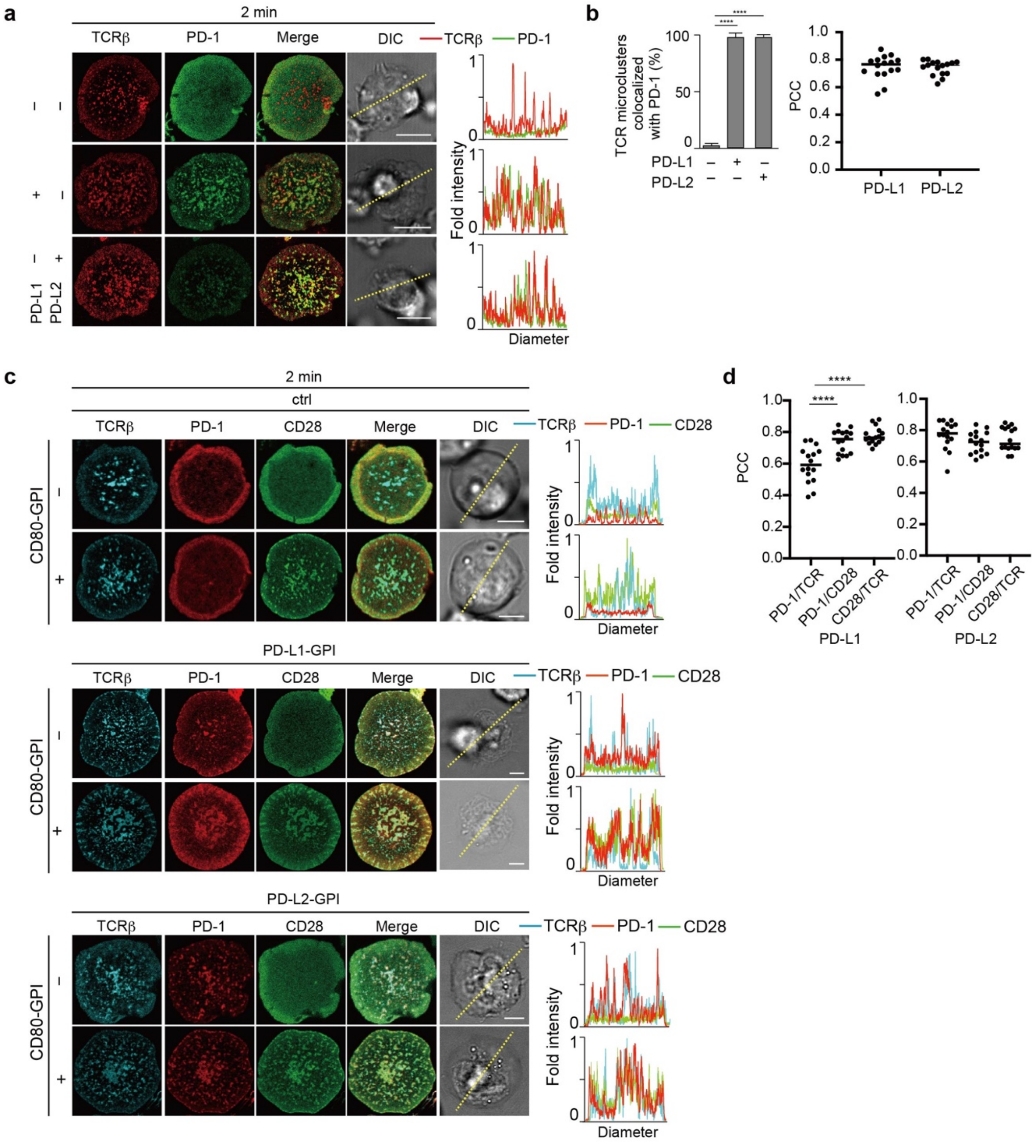

Supplementary Fig. 3 Comparison of the colocalization of PD-1 at TCR microclusters in the absence or presence of CD28–CD80 binding.

a 2D12 transduced with mPD-1–EGFP (green) were prestained with DyLight 650-labeled H57 Fab (red) and real-time imaged as in Fig. 1a. Histograms show fold fluorescent intensities of TCRβ (red) and PD-1 (green) on the diagonal yellow lines in the DIC images. **b** The left graph shows the

percentage of TCR microclusters colocalized with PD-1 at 2 min after contact in (**a**) (n = 5). The right graphs show the scatter plot summarizing the Pearson's correlation coefficient (PCC) values in (**a**). PCC between PD-1 and TCR was calculated in the presence of mPD-L1–GPI (0.74± 0.05, mean ± SD) or mPD-L2–GPI (0.74± 0.05, mean ± SD) by 16 randomly plotted profiles on 8 cells. **c** 2D12 expressing both mPD-1–HaloTag (red) and mCD28–EGFP (green) were prestained with both DyLight 549-labeled H57 Fab (cyan) and HaloTag ligand–Stella 650 and real-time imaged as in Fig. 1a with the indicated combinations of mCD80–GPI (30/μm^2), mPD-L1–GPI (150/μm^2) and/or mPD-L2–GPI (150/μm^2). Histograms show fold fluorescent intensities of TCRβ (cyan), PD-1 (red) or CD28 (green) on the diagonal yellow lines in the DIC images. **d** The graphs shows the scatter plot summarizing the PCC values in (**c**). PCC was calculated between PD-1/TCR (0.59± 0.05, mean ± SD), PD-1/CD28 (0.74± 0.05, mean ± SD) or CD28/TCR (0.59± 0.05, mean ± SD) in the presence of mPD-L1–GPI (left), or between PD-1/TCR (0.77± 0.05, mean ± SD), PD-1/CD28 (0.72± 0.05, mean ± SD) or CD28/TCR (0.74± 0.05, mean ± SD) in the presence of mPD-L2–GPI (right) by 16 randomly plotted profiles on 8 cells. All data are representatives from two independent experiments. Bars, 5 μm. Error bars, SD. Statistical analysis was by an unpaired Student's *t*-test and one-way analysis of variance (ANOVA). ****$p<0.0001$.

Supplementary Fig. 4

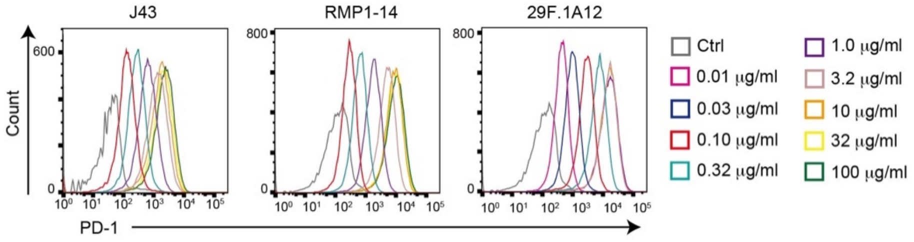

Supplementary Fig. 4 Flowcytometric analysis validating the avidity of each anti-mPD-1 clone.

2D12 expressing mPD-1 were stained on ice for 40 minutes by each anti-PD-1 clone at the indicated concentrations, secondary stained by APC-labelled anti-IgG and analyzed by FACS.

Supplementary Fig. 5

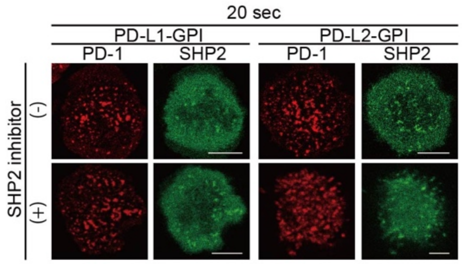

Supplementary Fig. 5 Formation of PD-1 microclusters on T cells treated with a SHP2 inhibitor.

2D12 transduced with both PD-1–HaloTag and EGFP–SHP2 in Fig. 3a were preincubated with the HaloTag ligand–Stella650 (red) and a SHP2 inhibitor, RMC4550, at a concentration of 50 μM for 2 hours. The cells were plated onto a planar bilayer with mPD-L1–GPI (left, 150/μm^2) or mPD-L2–GPI (right, 150/μm^2) and real-time imaged by confocal microscopy at 20 sec after T cell–bilayer contact in the absence (top) or presence (bottom) of the SHP2 inhibitor. A representative of two independent experiments is shown. Bars, 5 μm.

Supplementary Fig. 6

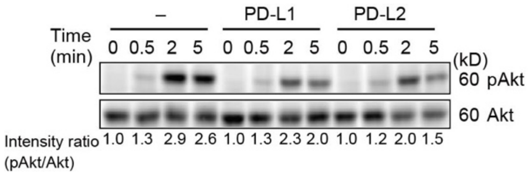

Supplementary Fig. 6 Suppression of the Akt signaling pathway by PD-1–PD-L1/L2 binding. Primary CD4$^+$ T cells transduced with mPD-1 were stimulated with $MCC_{88\text{-}103}$-prepulsed DC-1 cells not expressing (left) or expressing PD-L1 (middle) or PD-L2 (right) for the indicated times. The whole cell lysates were blotted for pAkt or Akt. The number below each line represents the intensity ratio, pAkt/Akt. A representative of three independent experiments is shown.

Supplementary Fig. 7

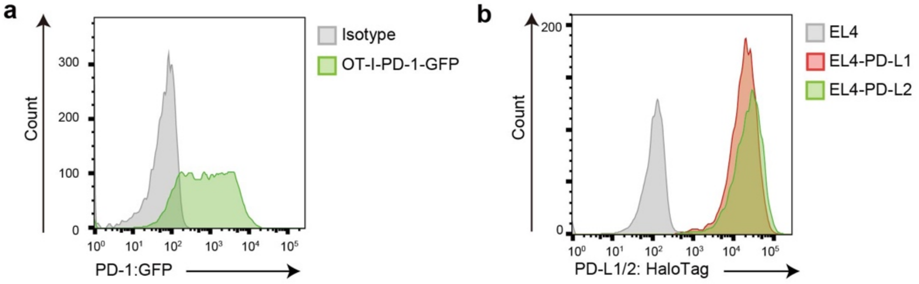

Supplementary Fig. 7   Normalization of the expression of PD-L1 and PD-L2 in EL-4 cells.

a $CD8^+$ T cells were purified from OT-I-Tg *Rag2*$^{-/-}$ mice, stimulated with immobilized anti-mCD3ε (2C11) and anti-mCD28 (PV-1) for 24 hours and retrovirally transduced with mPD-1–EGFP. The transduction efficiency of mPD-1–EGFP was analyzed by FACS. **b** To establish the target cells for OT-I Tg $CD8^+$ T cells, H-2K^{b+} EL4 cells were transduced by RLuc8 and further by HaloTag-tagged mPD-L1 or mPD-L2. EL4 cells expressing mPD-L1–HaloTag or mPD-L2–HaloTag were sorted by the same range of the intensity of HaloTag. The expression of mPD-L1– and mPD-L2–HaloTag in the equal level was confirmed by FACS.

Supplementary Fig. 8

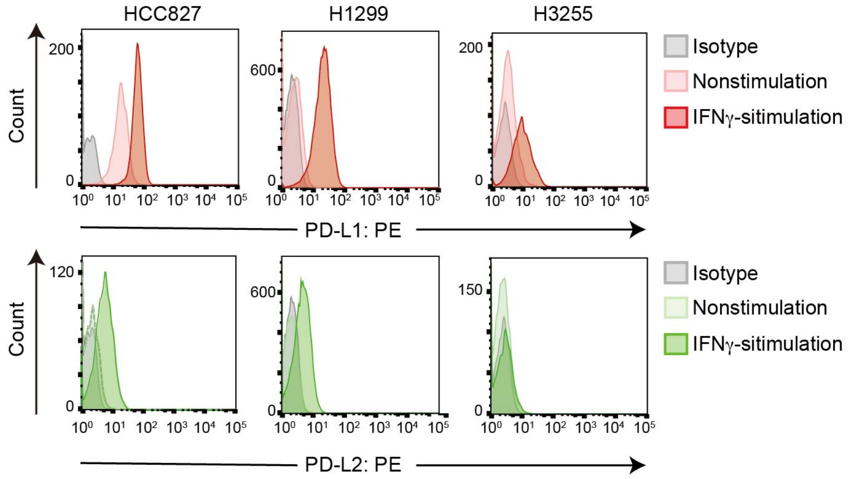

Supplementary Fig. 8 Expression of PD-L1 and PD-L2 in human lung cancer cell lines.

Human lung cancer cell lines, HCC827, H1299 and H3255, were stimulated with IFNγ at a concentration of 10 ng/mL for 48 hours. The cell surface expression of PD-L1 (top) and PD-L2 (bottom) was analyzed by FACS with PE-labeled anti-human (h) PD-L1 and anti-hPD-L2, respectively.

Supplementary Fig. 9

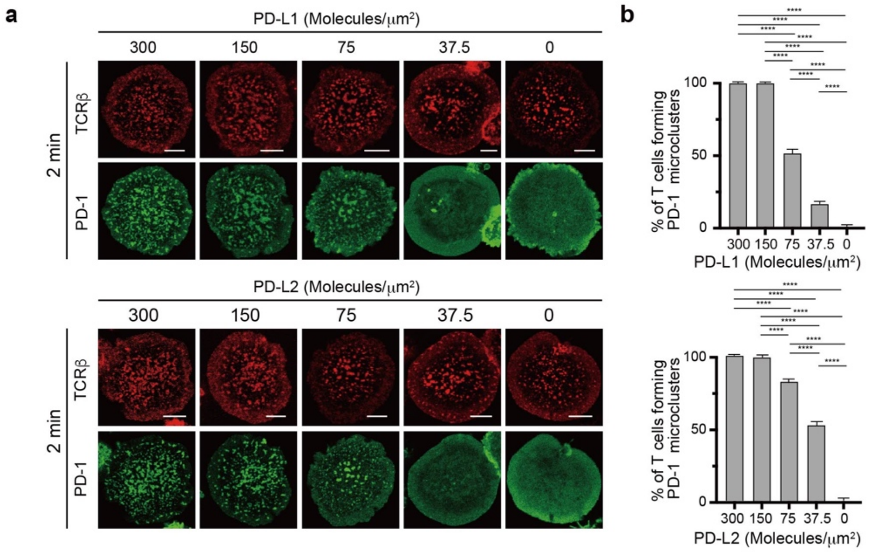

Supplementary Fig. 9 PD-L2 at low density forms more stable PD-1 microclusters than PD-L1.

2D12 expressing mPD-1–EGFP (green) were prestained with DyLight 650-labeled H57 Fab (red) and real-time imaged as in Figure 1a with different densities of mPD-L1–GPI (top) or mPD-L2–GPI (bottom) indicated. **b** The graphs show the percentage of T cells forming PD-1 microclusters in the presence of the indicated densities of PD-L1 (top) or PD-L2 (bottom) as in (**a**) (n = 30). Bars, 5 μm. Error bars, SD. A representative of three independent experiments is shown. Statistical analysis was by one-way analysis of variance (ANOVA). ****p<0.0001.

Supplementary Fig. 10

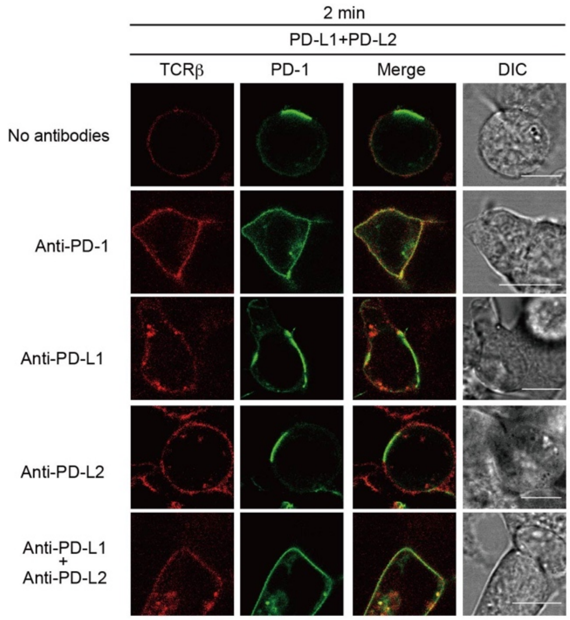

Supplementary Fig. 10 Accumulation of PD-1 at the T cell–APC interface is disrupted by the addition of antibodies for PD-1 or both PD-L1 and PD-L2.

2D12 expressing mPD-1–EGFP (green) were prestained with DyLight 650-labeled H57 Fab (red) and conjugated with DC-1 cells expressing both mPD-L1 and mPD-L2 in the absence (top) or presence of anti-PD-1 (row 2), anti-PD-L1 (row 3), anti-PD-L2 (row 4) or both anti-PD-L1 and anti-PD-L2 (bottom) as in Fig. 2a. The real-time images were acquired by confocal microscopy at 2 min after T cell–APC contacts. A representative of two independent experiments is shown. Bars, 5 μm.

Supplementary Fig. 11

Fig. 3c

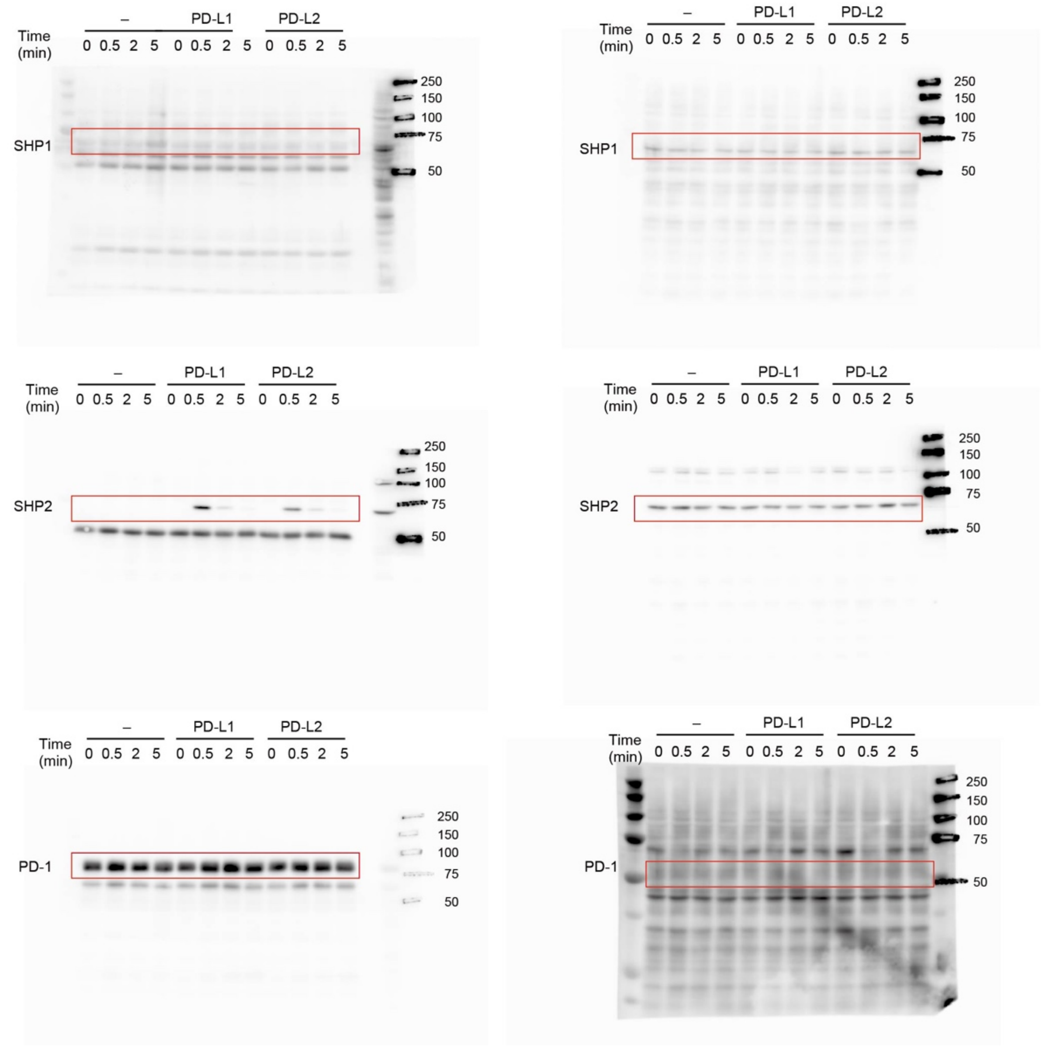

Supplementary Fig. 11 Full immunoprecipitation blot images corresponding to Fig. 3c.

Supplementary Fig. 12

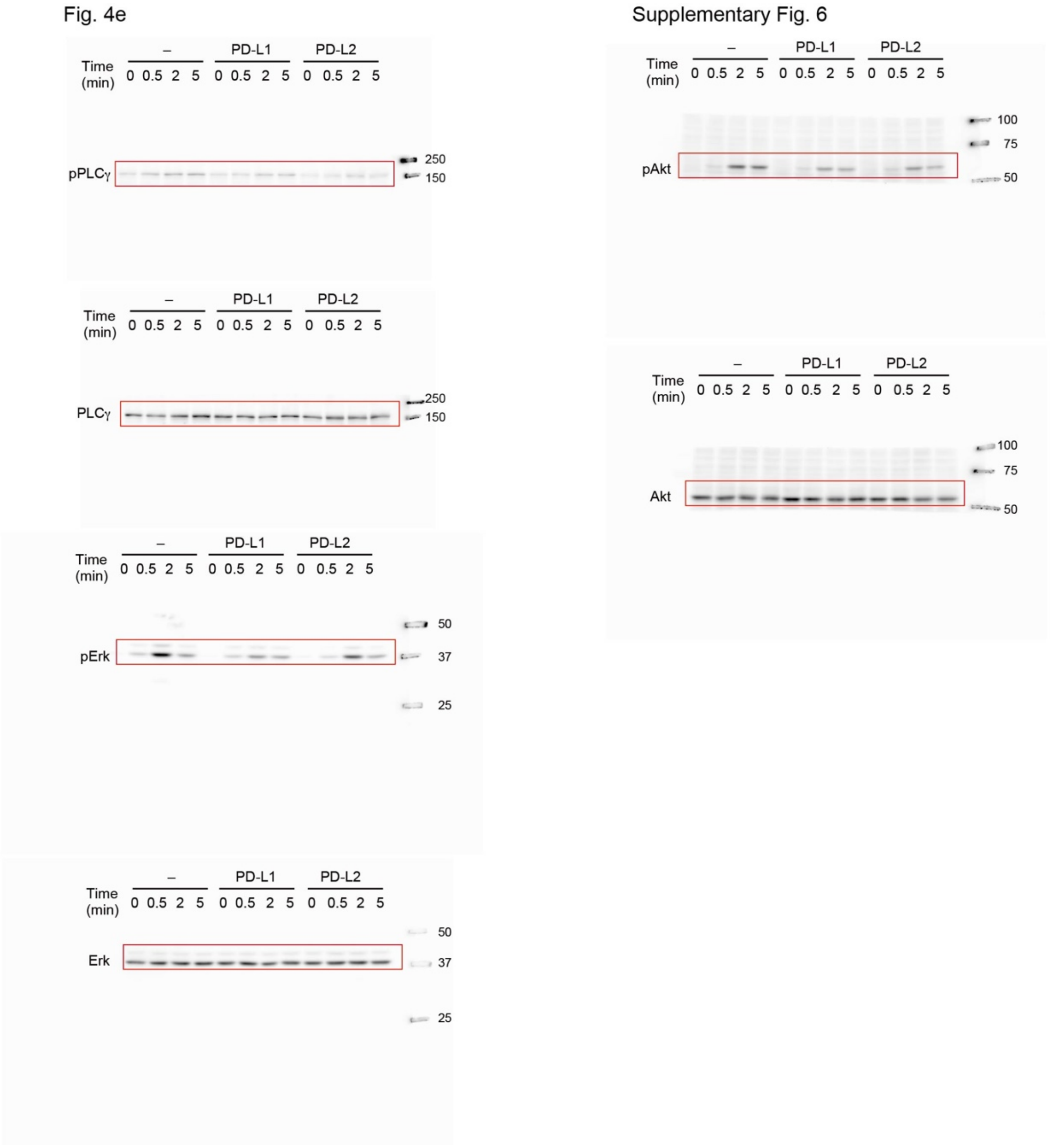

Supplementary Fig. 12 Full Western blot images corresponding to Fig. 4e and Supplementary Fig. 6.
