# Supplementary material for: PD-L2 suppresses T cell signaling via coinhibitory microcluster formation and SHP2 phosphatase recruitment
Source: Commun Biol. 2021 May 14;4:581. doi: 10.1038/s42003-021-02111-3 (PMC8121797; doi:10.1038/s42003-021-02111-3)
Supplement: Supplementary file 3 — Description of Additional Supplementary Files [file 42003_2021_2111_MOESM3_ESM.pdf]

Description of Additional Supplementary Files

File name: Supplementary Movie 1

Description: PD-1 forms microclusters in the presence of PD-L1 or PD-L2.

AND-Tg *Pdcd1*$^{-/-}$ $CD4^+$ T cells were introduced by mPD-1–EGFP in the equal level of endogenous PD-1, plated onto an $MCC_{88\text{-}103}$-prepulsed planar bilayer containing I-E^k–GPI and ICAM-1–GPI without (left) or with mPD-L1–GPI (middle) or mPD-L2–GPI (right) and imaged by TIRF microscopy at every 2.5 s. Bars, 5 µm. A representative of three independent experiments is shown.

File name: Supplementary Data 1

Description: Source data for figures and graphs
