# Supplementary material for: PD-L2 suppresses T cell signaling via coinhibitory microcluster formation and SHP2 phosphatase recruitment
Source: Commun Biol. 2021 May 14;4:581. doi: 10.1038/s42003-021-02111-3 (PMC8121797; doi:10.1038/s42003-021-02111-3)
Supplement: Supplementary file 6 — Reporting Summary [file 42003_2021_2111_MOESM6_ESM.pdf]

nature research

Corresponding author(s): Tadashi Yokosuka, COMMSBIO-20-3492B

Last updated by author(s): Mar 28, 2021

Reporting Summary

Nature Research wishes to improve the reproducibility of the work that we publish. This form provides structure for consistency and transparency in reporting. For further information on Nature Research policies, see our Editorial Policies and the Editorial Policy Checklist.

Statistics

For all statistical analyses, confirm that the following items are present in the figure legend, table legend, main text, or Methods section.

n/a	Confirmed	
☐	☒	The exact sample size (n) for each experimental group/condition, given as a discrete number and unit of measurement
☐	☒	A statement on whether measurements were taken from distinct samples or whether the same sample was measured repeatedly
☐	☒	The statistical test(s) used AND whether they are one- or two-sided<br>*Only common tests should be described solely by name; describe more complex techniques in the Methods section.*
☐	☒	A description of all covariates tested
☐	☒	A description of any assumptions or corrections, such as tests of normality and adjustment for multiple comparisons
☐	☒	A full description of the statistical parameters including central tendency (e.g. means) or other basic estimates (e.g. regression coefficient) AND variation (e.g. standard deviation) or associated estimates of uncertainty (e.g. confidence intervals)
☐	☒	For null hypothesis testing, the test statistic (e.g. F, t, r) with confidence intervals, effect sizes, degrees of freedom and P value noted<br>*Give P values as exact values whenever suitable.*
☒	☐	For Bayesian analysis, information on the choice of priors and Markov chain Monte Carlo settings
☒	☐	For hierarchical and complex designs, identification of the appropriate level for tests and full reporting of outcomes
☒	☐	Estimates of effect sizes (e.g. Cohen's d, Pearson's r), indicating how they were calculated

Our web collection on statistics for biologists contains articles on many of the points above.

Software and code

Policy information about availability of computer code

Data collection	No software was used.
Data analysis	Prism v9.0.1 (GraphPad), FlowJo v10.5.0 (TreeStar), Fiji v2.0

For manuscripts utilizing custom algorithms or software that are central to the research but not yet described in published literature, software must be made available to editors and reviewers. We strongly encourage code deposition in a community repository (e.g. GitHub). See the Nature Research guidelines for submitting code & software for further information.

Data

Policy information about availability of data

All manuscripts must include a data availability statement. This statement should provide the following information, where applicable:
- Accession codes, unique identifiers, or web links for publicly available datasets
- A list of figures that have associated raw data
- A description of any restrictions on data availability

Source data for graphs in the main figures and supplementary figures are available in the Supplementary Data. All data supporting the conclusions are included in the manuscript, supplementary figures and a movie, or are available from the corresponding author upon reasonable request.

Field-specific reporting

Life sciences study design

All studies must disclose on these points even when the disclosure is negative.

Sample size	No sample size was calculated in this study.
Data exclusions	No data were excluded form the analyses.
Replication	All experiments were repeated at indicated time in each figure legend and the results were repeatable.
Randomization	The cells were randomly selected in the analysis of imaging data.
Blinding	No blinded analyses were performed.

Reporting for specific materials, systems and methods

We require information from authors about some types of materials, experimental systems and methods used in many studies. Here, indicate whether each material, system or method listed is relevant to your study. If you are not sure if a list item applies to your research, read the appropriate section before selecting a response.

Materials & experimental systems

n/a	Involved in the study	
☐	☒	Antibodies
☐	☒	Eukaryotic cell lines
☒	☐	Palaeontology and archaeology
☐	☒	Animals and other organisms
☒	☐	Human research participants
☒	☐	Clinical data
☒	☐	Dual use research of concern

Methods

n/a	Involved in the study	
☒	☐	ChIP-seq
☐	☒	Flow cytometry
☒	☐	MRI-based neuroimaging

Antibodies

Antibodies used

1. PE–anti-mCD80 (16-10A1), PE–anti-I-A/I-E (M5/114.15.2), PE–anti-hPD-L1 (MIH1), PE–anti-hPD-L2 (MIH18), FITC– or PE–isotype-matched control IgG from eBioscience,HRP-anti-mouse IgG polyclonal Abs from Cappel for flow cytometry.

2. FITC– or PE–anti-mPD-L1 (MIH5), FITC– or PE–anti-mPD-L2 (MIH37), PE–anti-mPD-L2 (TY25),anti-mPD-1 (J43 and 29F.1A12) from Bio X Cell, anti-mPD-1 (RMP1-14) from BioLegend, Alexa Fluor 647-labeled anti-pCD3z (K25-407.69), Alexa Fluor 647-labeled anti-pSLP-76 (J141-668.36.58) from BD, anti-TCRb (H57-597) by RT. Kubo (Cytel Corp., CA, USA), anti-I-Ek (14-4-4) and anti-ICAM-1 (YN1/1.7.4) by ML. Dustin (Univ. of Oxford, UK), Alexa Fluor 647-labeled anti-rat IgG (H+L), Alexa Fluor 647-labeled anti-hamster IgG (H+L), DyLight 650 and 549 labeling kits from Thermo Fisher Scientific for imaging analysis.

3. Rabbit polyclonal anti-SHP1 (C-19) and anti-SHP2 (C-18), goat polyclonal anti–PD-1 (E-18), HRP–labeled polyclonal anti-mouse IgG from Santa Cruz Biotechnology Inc, anti-Erk, anti-pErk, anti-PLCg, anti-pPLCg, anti-Akt, anti-pAkt from Cell Signaling Technology for immunoprecipitation and western blotting.

4. Anti-IL-2 (JES6-1A12), biotin-labeled anti-IL-2 (JES6-5H4) from eBioscience, anti-IFNg (RA-6A2), biotin-labeled anti-IFNg (XMG1.2), HRP-anti-rabbit IgG from Cell Signaling Technology for ELISA.

Validation

Each antibody was validated by the manufacturers and its data are are available on their website.

Eukaryotic cell lines

Policy information about cell lines

Cell line source(s)

The DC-1 fibroblast cells expressing I-Ek and ICAM-1 were provided by J. Kaye (Cedars-Sinai Medical Center Los Angeles, CA). PLAT-E, the retrovirus packaging cell line, was provided by G. Nolan (Stanford University, Stanford, CA). Human lung cancer cell lines HCC827, H1299 and H3255 were purchased from ATCC. BHK and EL-4 cells were purchased from ATCC. The T cell hybridoma expressing the AND-TCR (AND hybridoma) was established previously. The B cell hybridoma producing anti-CD28 (PV-1) was provided by R. Abe (Tokyo University of Science, Noda, Japan).

Authentication

All cells were purchased from companies or kindly provided by researchers with agreement.

Mycoplasma contamination	All cell lines were tested negative for Mycoplasma contamination.
Commonly misidentified lines (See ICLAC register)	None.

Animals and other organisms

Policy information about studies involving animals; ARRIVE guidelines recommended for reporting animal research

Laboratory animals	The AND-TCR-Tg mouse was provided by Dr. SM. Hedrick (University of California San Diego, San Diego, CA), Rag2-/- mice by Dr. F. Alt (Boston Children's Hospital, Boston, MA), OT-I-TCR-Tg Rag2-/- mice by Dr. W. Health (University of Melbourne, Melbourne, Australia); Pdcd1-/- mice (RIKEN BRC).
Wild animals	n/a
Field-collected samples	n/a
Ethics oversight	All the murine experiments in this study were performed in accordance with a protocol approved by the Animal Care and Use Committee of Tokyo Medical University (H30-0044, H31-0065, R2-0001).

Note that full information on the approval of the study protocol must also be provided in the manuscript.

Flow Cytometry

Plots

Confirm that:

- [x] The axis labels state the marker and fluorochrome used (e.g. CD4-FITC).
- [ ] The axis scales are clearly visible. Include numbers along axes only for bottom left plot of group (a 'group' is an analysis of identical markers).
- [ ] All plots are contour plots with outliers or pseudocolor plots.
- [x] A numerical value for number of cells or percentage (with statistics) is provided.

Methodology

Sample preparation	The cells were suspended with FACS buffer (2% FCS/PBS) and stained with fluorescence-conjugated antibodies. Stained cells were washed twice with FACS buffer and then analyzed.
Instrument	Canto II (BD), SH800 (Sony)
Software	FlowJo v10.5.0
Cell population abundance	100000 events in FSC/SSC parameters were measured to analyze.
Gating strategy	Live cells were gated by FSC/SSC and doublet cells were removed. Then target cells were counted or sorted for fluorescence-positive area.

- [x] Tick this box to confirm that a figure exemplifying the gating strategy is provided in the Supplementary Information.
